# External fixator immobilisation of a pedicled groin flap

**DOI:** 10.1308/rcsann.2014.96.1.75

**Published:** 2014-01

**Authors:** Y Sheena, R McCulloch, D Evriviades

**Affiliations:** Royal Centre for Defence Medicine,UK

## BACKGROUND

Pedicled flaps can still be a useful part of the reconstructive surgical toolbox. Their stabilisation is crucial to prevent shearing forces causing flap failure. This can be challenging using traditional dressings so different stabilisation methods have been described including plaster of Paris and topical negative pressure dressings. These are disadvantaged by limiting flap observations and wound care. External fixation was first described for this indication nearly half a century ago^1–3^ but recently, we could only find a Chinese case series using this technique published, with only its abstract in English.^4^ We aim to illustrate its effective use in giving an excellent immobilisation, flap care and outcome.

## TECHNIQUE

**Figure 1:**
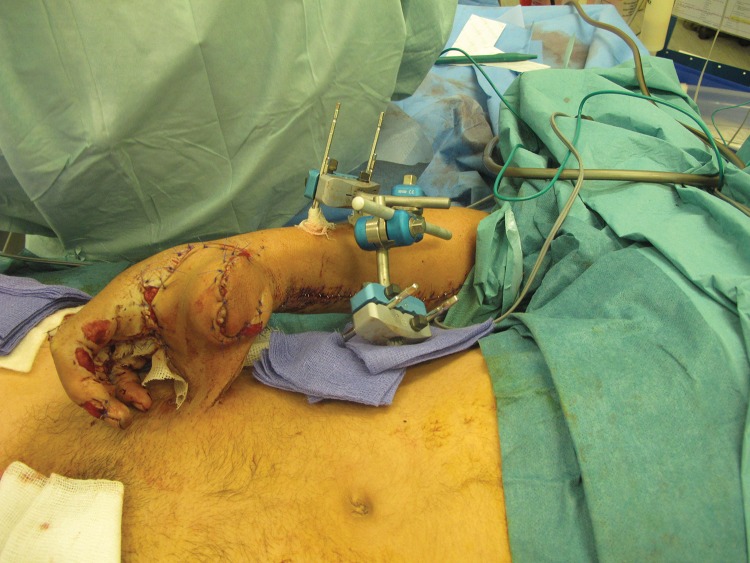
The external fixator at the end of the procedure showing the pedicled ipsilateral groin flap stabilised to the right palm

**Figure 2:**
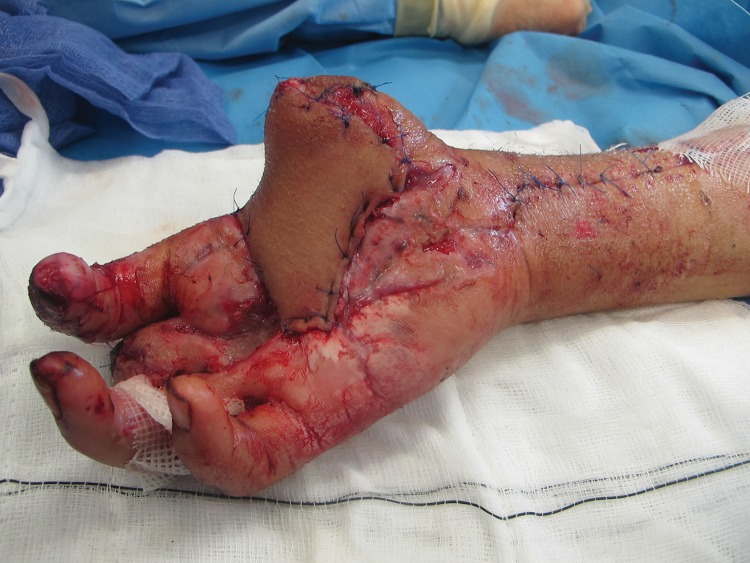
Result at day 14 after the external fixator was removed and the flap divided

In order to stabilise an ipsilateral pedicled groin flap to resurface a palmar hand defect, a Hoffmann style external fixator was used with pins inserted into the ipsilateral distal radius and anterior superior iliac spine (Fig 1). The frame can be adjusted to allow a small amount of movement to minimise joint stiffness. The external fixator is removed and the flap divided at two weeks (Fig 2).

## DISCUSSION

External fixation to immobilise pedicled flaps provides robust stabilisation of the flap with excellent access for observation and wound care. Ease of application and removal facilitates flap management as well as definitive patient rehabilitation and hand therapy. Potential risks of bone fracture, muscle cramps and pin site infection can be minimised by appropriate placement/adjustment, wound care and patient counselling.^5^ In our experience, external fixation flap stabilisation is safe and effective, and we hope other surgeons will publish their outcomes using this technique.

## DECLARATION

Consent was secured from the patient and the UK Ministry of Defence for publishing this report and the clinical photographs.
